# The value of nurse-led anthropometric and oropharyngeal measurements combined with STOP-Bang questionnaire in screening for obstructive sleep apnea in patients with acute coronary syndrome: a prospective cohort study

**DOI:** 10.1186/s12890-022-02200-x

**Published:** 2022-11-03

**Authors:** Zexuan Li, Hua Miao, Siyu Zhang, Jingyao Fan, Yan Yan, Wei Gong, Wen Zheng, Xiao Wang, Bin Que, Hui Ai, Lixin Zhang, Shaoping Nie

**Affiliations:** 1 Center for Coronary Artery Disease, Division of Cardiology, Beijing Anzhen Hospital, Capital Medical University, No. 2 Anzhen Road, Chaoyang District, Beijing, 100029 China; 2 National Clinical Research Center for Cardiovascular Diseases, Beijing, China; 3 Qingdao Special Servicemen Recuperation Center of PLA Navy, Qingdao, Shandong China

**Keywords:** Anthropometric measurements, Oropharyngeal, STOP-Bang questionnaire, Obstructive sleep apnea, Acute coronary syndrome

## Abstract

**Background:**

Obstructive sleep apnea (OSA) is a modifiable risk factor for acute coronary syndrome (ACS), with high prevalence but low diagnostic rates. Therefore, it is particularly important to develop strategies for better screening for OSA in newly admitted ACS patients.

**Methods:**

From March 2017 to October 2019, consecutive eligible patients with ACS underwent cardiorespiratory polygraphy during hospitalization. OSA was defined as an apnea–hypopnea index (AHI) ≥ 15 events/h. All anthropometric and oropharyngeal parameters are measured by specialist nurses.

**Results:**

Finally, 761 ACS patients were recruited in the present study. Prevalence of moderate/severe OSA was 53.2% based on diagnostic criteria of AHI ≥ 15. Correlation analysis illustrated that AHI was positively correlated with anthropometric characteristics. In the multivariate model, only micrognathia (OR 2.02, 95% CI 1.02–4.00, *P* = 0.044), waist circumference (OR 1.08, 95% CI 1.04–1.11, *P* < 0.001), and STOP-BANG Questionnaire (SBQ) score (OR 1.45, 95% CI 1.27–1.66, *P* < 0.001) were independently associated with the prevalence of OSA. Receiver operating characteristic curve (ROC) analysis showed that the area under curve (AUC) of multivariable joint diagnosis (waist circumference, micrognathia combined with SBQ) was significantly better than the AUC of Epworth Sleepiness Scale (ESS) and SBQ (*p* < 0.0001 and *p* = 0.0002, respectively), and the results showed that AUC was 0.728. Under the optimal truncation value, the sensitivity was 73%, and the specificity was 61%, which was higher than the single index. Finally, we also constructed a nomogram model based on multiple logistic regression, to easily determine the probability of OSA in ACS patients.

**Conclusions:**

The new screening tool has greater power than single questionnaire or measurements in screening of OSA among ACS patients.

**Trial registration:**

Clinicaltrials.gov identifier NCT03362385, registered December 5, 2017.

## Background

Obstructive sleep apnea (OSA) is a complex and heterogeneous common chronic disease, affecting 34% of men and 17% of women. In addition, OSA is a modifiable risk factor for acute coronary syndrome (ACS) with a high prevalence but low diagnosis rate [1]. OSA was linked to a higher risk of subsequent events in hospitalized ACS patients, especially in female patients [2]. Moderate/severe OSA is associated with increased all-cause and CAD mortality [3]. Most clinical studies have shown that the incidence of breathing disorders during sleep in acute myocardial infarction patients is as high as 60% [4]. In addition, patients with OSA and ACS have significantly increased major adverse cardiac events (MACEs) and poor prognosis [5–7].

Epidemiological studies have shown that many factors are good predictors of OSA, but it is reasonable to assume that these predictors may vary depending on the particular population studied [8–11]. It is well known that continuous overnight polysomnography (PSG) is the gold standard for diagnosing OSA [3]. However, PSG requires patients to be hospitalized for examination, which is time-consuming and labor-intensive. During the examination, many electrodes are worn, and changes in sleeping position and environment can easily cause psychological and physical discomfort to patients, even cause insomnia and interfere with the accuracy of PSG results [12]. The Epworth Sleepiness Scale (ESS) and the STOP-BANG Questionnaire (SBQ) are commonly used in clinical primary screening questionnaires [13, 14], but as evaluation criteria for patients' subjective judgment, their primary screening diagnosis is limited and subjective. Therefore, it is particularly important to develop strategies for better screening for sleep apnea in newly admitted ACS patients [15].

Anthropometric and oropharyngeal parameters such as height, weight, waist, neck and hip circumferences, enlarged tonsils, upper airway obstruction, and micrognathia are easily obtained by nurses after admission for ACS patients, which are more objective than sleep questionnaires. It is proposed to use a nurse-led, readily available anthropometric and oropharyngeal parameters combined with sleep questionnaires to improve the diagnosis of OSA in patients with ACS. The primary screening of moderate/severe OSA should be performed on the admitted ACS patients, and the patients after screening can be directly referred to the sleep center for PSG to avoid the waste of medical resources.

## Methods

### Study design and subjects

The OSA-ACS project (NCT03362385) is a prospective, observational, single-center study to assess the association of OSA with cardiovascular outcomes of patients with ACS. The study design has been described previously [6, 16]. Between March 2017 and October 2019, anthropometric and oropharyngeal parameters were collected in enrolled patients who had portable cardiorespiratory polygraphy. Inclusion criteria: (1) aged 18–85 years; (2) discharged with a diagnosis of ACS, including ST-segment elevation myocardial infarction (STEMI), non-ST-segment elevation myocardial infarction (NSTEMI), and unstable angina (UA); (3) Sign the informed consent; (4) Completion of sleep questionnaires (ESS and STOP-BANG questionnaires) while awake. Exclusion criteria: (1) previous or ongoing continuous positive airway pressure, (CPAP) treatment; (2) intolerance, unable to complete sleep monitoring examination; (3) central sleep apnea [central event ratio ≥ 50% of all events and central sleep apnea hypopnea index (apnea hypopnea index), AHI) ≥ 10 times/h]. This study protocol was approved by the Ethics Committee of Beijing Anzhen Hospital Affiliated to Capital Medical University (2,013,025). This study conformed to the Declaration of Helsinki. Finally, 761 ACS patients were recruited in the present study.

### Cardiorespiratory polygraphy

After ACS patients were admitted to hospital in stable condition, all enrolled patients were examined with portable cardiorespiratory polygraphy (Apnea Link Air, ResMed, Australia). The monitoring contents include nasal airflow, chest and abdominal movement, heart rate, heart rhythm, snoring sound and body position, the lowest and average arterial oxygen saturation at night (SaO_2_), and the percentage of SaO_2_ < 90% of the total recording time (referred to as SaO_2_ < 90% of the time). All sleep studies were manually double scored by independent sleep research staff who had no knowledge of the clinical features and confirmed by senior research staff in cases of discrepancy. All data were reviewed to avoid omissions or miscalculations. Apnea was defined as complete cessation of oronasal airflow for ≥ 10 s, and hypopnea was defined as oronasal airflow drop ≥ 30% for ≥ 10 s, and combined with SaO_2_ > 4%. Effective monitoring refers to the recording time ≥ 3 h. The apnea–hypopnea index (AHI) was defined as the number of apneas or hypopneas per hour of total recording time. All sleep studies were scored according to the American Academy of Sleep Medicine (AASM)2007 guidelines. The selected patients were divided into moderate/severe OSA group (AHI ≥ 15 times/h) and no/mild OSA group (AHI < 15 times/h).

### Anthropometric and oropharyngeal measurements

Anthropometric and oropharyngeal parameters are measured and collected by the trained specialist nurse from the ward clinical team, who was not always the same member of staff. The nurses must have received specific clinical instruction on anthropometry and oropharyngeal measurements and be aware of the following measurement protocols. Body mass index (BMI) was calculated as weight (in kilograms) divided by height (in meters) squared. Neck circumference was measured at the level of the cricothyroid membrane. Waist circumference (WC) was measured at horizontal girth across the center of the navel, and hip circumference was measured at the level of the two bony prominences in front of the hips. Micrognathia is considered to be a patient with a pronounced mandibular retraction when viewed from the side, presenting a bird-like appearance. The narrowing of the upper airway is judged by the nurse according to the Mallampati score (Class I: complete visualization of the soft palate, Class II: complete visualization of the uvula, Class III: visualization only the base of the uvula, Class IV: soft palate is not visible at all.) Class III and IV are considered airway obstruction and are at risk for OSA. According to the classification of antiadoncus, tonsillar enlargement not exceeding the pharyngopalatine arch was denoted as grade I, tonsillar enlargement exceeding the pharyngopalatine arch was denoted as grade II, and tonsillar enlargement to the midline of the posterior pharyngeal wall was denoted as grade III. To minimize the influence of subjective judgments on the reliability of the results, the otolaryngologist developed a measurement protocol and picture in which the judgment of upper airway obstruction and micrognathia had to be guided by a doctor (ZL) and measured by an experienced senior nurse (HM or SZ). When there was disagreement between them, the patient was evaluated by a senior cardiologist (SN) and a consensus was reached.

### Sleep screening questionnaires

When the patient is full of energy, a trained specialist nurse will explain the relevant items and scoring methods of the sleep questionnaires (ESS and SBQ) to the patient, and the scale score will be calculated after filling in the scale under supervision. The ESS consists of the following 8 items: sitting and reading, watching TV, being inactive in a public place (such as a theater or a meeting), being in the car as a passenger for an hour straight, lying down in the car and resting in the afternoon, if conditions permit, sitting talking to someone, sitting quietly after lunch, not drinking, in a car, stuck in traffic for a few minutes. Each item is scaled from 0 to 3, with 0 is never dozing off, 1 is slight chance of dozing off, 2 is moderate chance of dozing off, and 3 is high chance of dozing off, the total score is 0–24 points, the score ≥ 10 points is the risk of OSA. The STOP-BANG questionnaire (SBQ) includes 8 items, 6 of which are related to snoring, daytime sleepiness, sleep apnea, hypertension, age and gender are self-administered questions, while 2 items of patient BMI and neck circumference are measured by nurses. Answering yes to three or more items is categorized as high risk for OSA.

### Statistical analysis

Continuous variables were presented as mean ± standard deviation (SD) or median (first and third quartiles) and were compared by Student’s t-test or Mann–Whitney U test. Categorical variables were exhibited as the number (percentage) and were compared using chi-square test or Fisher’s exact test. The correlations between Anthropometric Characteristics and AHI were determined by Spearman’s correlation analysis. Binary logistic regression analysis was performed to explore the association of anthropometric, oropharyngeal parameters, and sleep questionnaires (continuous scores) with OSA. The sensitivity, specificity, positive predictive value, and negative predictive value of anthropometric characteristics combined with sleep questionnaires were calculated by the four-grid method. The ROC curve was drawn to analyze the diagnostic value of anthropometric and oropharyngeal measurements combined with STOP-Bang in screening for OSA in patients with ACS. For the indicators with statistical differences in multivariate logistic analysis, they were used as predictors, and the Nomogram model based on multivariate logistic regression was established using the R (http://www.R-project.org) and Empower Stats software (www.empowerstats.com, X&Y solutions, Inc. Boston MA). All tests were 2-sided, and the value of *P* < 0.05 was considered statistically significant. Statistical analysis was performed with SPSS (version 26.0 IBM SPSS Inc, Armonk, NY).

## Results

### Clinical characteristics and demographics

**Fig. 1:**
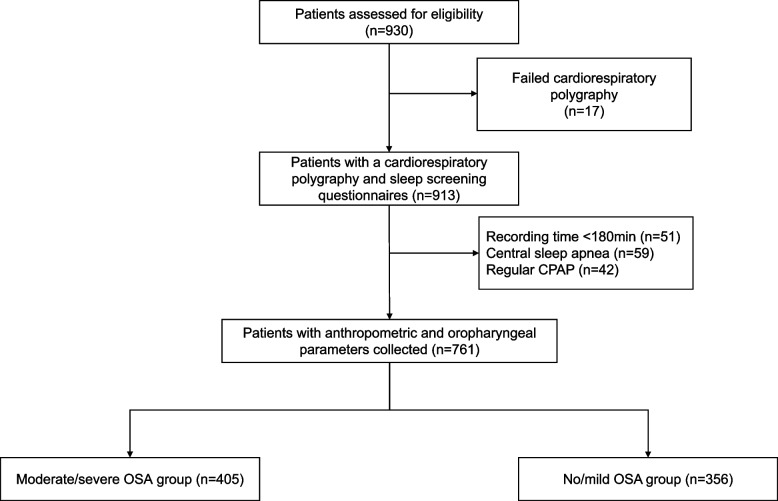
Study flowchart. CPAP: continuous positive airway pressure; OSA: obstructive sleep apnea

Of 930 eligible subjects of the OSA-ACS project, 761 subjects had complete data collected and were included in the final analysis (Fig. 1). The baseline characteristics and demographics of subjects with moderate/severe OSA and no/mild OSA are shown in Table 1. The mean patient age was 56.1 ± 10.6 years, and 85.4% were male. Patients with moderate/severe OSA were more likely to be male and current smokers and had significantly higher anthropometric characteristics (body mass index, waist, hip, and neck circumferences) compared with those with no/mild OSA. In addition, micrognathia and upper airway obstruction were more frequent in patients with moderate to severe OSA. Medical history was comparable between two groups, except hypertension, which was more frequent in the moderate/severe OSA group. Other baseline information was generally well matched between moderate/severe OSA group and no/mild OSA groups (Table 1).

**Table 1:** Characteristics of patients in the study

Variables	Total (*n* = 761)	Moderate/severe OSA (*n* = 405)	No/mild OSA (*n* = 356)	P
Age (years)	56.1 ± 10.6	56.1 ± 10.9	56.1 ± 10.1	0.993
Male (%)	650(85.4)	356(87.9)	294(82.6)	0.038
BMI (kg/m^2^)	27.2 ± 3.7	28.3 ± 3.6	26.0 ± 3.33	< 0.001
Neck circumference (cm)	41(38–43)	41.5(39–44)	40(37.5–42)	< 0.001
Waist circumference (cm)	99(94–106)	102(96–109)	96(91–102)	< 0.001
Hip circumference (cm)	101(97–106)	102(99–108)	100(96–104)	< 0.001
Micrognathia (%)	54(7.0)	40(9.9)	14(3.9)	0.001
Antiadoncus (%)	441(58)	238(58.8)	203(57)	0.627
Upper airway obstruction (%)	351(46.1)	203(50.1)	148(41.6)	0.018
Systolic BP (mm/Hg)	127(118–138)	127(119–139)	126(117–137)	0.291
Diastolic BP (mm/Hg)	77(77–85)	79(70–86)	75(70–82)	0.012
Hypertension (%)	489(64.3)	281(69.4)	208(58.4)	0.002
Hyperlipidemia (%)	254(33.4)	131(32.3)	123(34.6)	0.520
Diabetes mellitus (%)	231(30.4)	124(30.6)	107(30.1)	0.867
Current smoking (%)	337(44.3)	194(47.9)	143(40.2)	0.032
Prior stroke (%)	88(11.6)	50(12.3)	38(10.7)	0.472
Previous MI (%)	128(16.8)	66(16.3)	62(17.4)	0.680
Previous PCI (%)	166(21.8)	94(23.2)	72(20.2)	0.320
Previous CABG (%)	522(68.6)	284(70.1)	238(66.9)	0.332
LDL-cholesterol (mmol/L)	2.38(1.86–3.09)	2.41(1.92–3.05)	2.38(1.79–3.20)	0.770
HDL-cholesterol (mmol/L)	1.01(0.87–1.18)	0.98(0.86–1.16)	1.03(0.89–1.21)	0.017
Total cholesterol (mmol/L)	4.07(3.42–4.94)	4.08(3.5–4.85)	4.05(3.35–5.09)	0.951
Triglyceride (mmol/L)	1.52(1.08–2.19)	1.62(1.15–2.30)	1.43(1.03–2.05)	0.001
LVEF (%)	62(58–66)	62(57–66)	63(60–66)	0.172
hsCRP (mg/L)	1.79(0.76–5.1)	2.37(0.87–6.25)	1.22(0.52–3.31)	< 0.001
HbA1C (%)	6.1(5.6–7.0)	6.1(5.7–7.1)	6(5.6–6.9)	0.103
Fasting glucose (mmol/L)	6(5.37–7.51)	6.10(5.35–7.56)	5.89(5.38–7.39)	0.264
AHI (events/h)	16.3(8.4–29.4)	28.2(20.3–42.8)	8.0(4.5–11.1)	< 0.001
ODI (events/h)	16.6(9.5–27.6)	26.5(19.4–38.7)	9.1(5.7–12.1)	< 0.001
Minimum SaO_2_ (%)	86(82–88)	83(78–86)	88(85–89)	< 0.001
Time with SaO_2_ < 90% (%)	3(1–12)	7(2–20)	1(0.2–3)	< 0.001
ESS	7(3–11)	8(4–12)	6(3–10)	< 0.001
SBQ	4(4–5)	4(3–5)	3(3–4)	< 0.001

Data are presented as mean ± SD, median (first quartile: third quartile), or n (%)

*AHI* Apnea–hypopnea index, *BMI* Body mass index, *BP* Blood pressure, *ESS* Epworth sleepiness scale, *HbA1c* Glycated hemoglobin, *HDL* High-density lipoprotein, *hsCRP* High-sensitivity C-reactive protein, *LDL* Low-density lipoprotein, *LVEF* Left ventricular ejection fraction, *MI* Myocardial infarction, *ODI* Oxygen desaturation index, *OSA* Obstructive sleep apnea, *PCI* Percutaneous coronary intervention, *SaO*_*2*_ Arterial oxygen saturation, *SBQ* Stop-bang questionnaire

### Results of cardiorespiratory polygraphy

Prevalence of moderate/severe OSA was 53.2% based on diagnostic criteria of AHI ≥ 15. Patients with moderate/severe OSA exhibited lower minimum oxygen saturation, more time of SaO_2_ < 90%, higher AHI, oxygen desaturation index (ODI) and higher sleep questionnaire (ESS and SBQ) scores than those no/mild OSA. Detailed information is described in Table 1.

### Correlation of anthropometric measurements and sleep questionnaires with AHI

To evaluate the association between anthropometric characteristics, sleep questionnaires and AHI, correlation analysis were performed (Table 2). Spearman’s correlation analysis illustrated that AHI was positively correlated with the aforementioned variables. The difference was statistically significant (*P* < 0.001). Among these variables, WC (*r* = 0.357, *P* < 0.001), and SBQ (*r* = 0.378, *P* < 0.001) had the highest correlation coefficients.

**Table 2:** Correlation of nurse-led anthropometric characteristics and sleep questionnaires with AHI

Variables	AHI	
	*r*	*P*
BMI	0.351	< 0.001
Neck circumference	0.301	< 0.001
Waist circumference	0.357	< 0.001
Hip circumference	0.227	< 0.001
ESS	0.154	< 0.001
SBQ	0.378	< 0.001

*BMI* Body mass index, *ESS* Epworth sleepiness scale, *SBQ* Stop-bang questionnaire

### Association of anthropometric, oropharyngeal parameters, and sleep questionnaires with moderate/severe OSA

To evaluate the association between variables and OSA, univariate and multivariate logistic regression analyses were performed. In univariate logistic regression, we found that WC, hip circumference, micrognathia, upper airway obstruction, ESS and SBQ score were significantly associated with the prevalence of OSA. In the multivariate model, only micrognathia (OR 2.019, 95% CI 1.020–4.000, *P* = 0.044), WC (OR 1.075, 95% CI 1.044–1.108, *P* < 0.001), and SBQ score (OR 1.451, 95% CI 1.267–1.661, *P* < 0.001) were independently associated with the prevalence of moderate/severe OSA (Table 3). BMI and neck circumference were excluded from the model due to their high multicollinearity with SBQ.

**Table 3:** Univariate and multivariate logistics regression exploring the association of anthropometric, oropharyngeal characteristics, and sleep questionnaires (continuous scores) with moderate/severe OSA

Variables	Univariate			Multivariate		
	OR	95% CI	*P*	OR	95% CI	*P*
Waist circumference	1.053	0.612–1.314	0.004	1.075	1.044–1.108	< 0.001
Hip circumference	0.958	0.922–0.996	0.030	0.976	0.940–1.012	0.188
Micrognathia	2.381	1.262–4.493	0.002	2.019	1.020–4.000	0.044
Antiadoncus	1.074	0.805–1.433	0.672	-	-	-
Upper airway obstruction	1.385	1.039–1.846	0.027	1.099	0.800–1.510	0.559
ESS	1.060	1.029–1.092	< 0.001	1.020	0.986–1.055	0.260
SBQ	1.473	1.292–1.679	0.001	1.451	1.267–1.661	< 0.001

*ESS* Epworth sleepiness scale, *SBQ* Stop-bang questionnaire

### Diagnostic accuracy for anthropometric and oropharyngeal parameters combined with sleep questionnaire to detect moderate/severe OSA

**Fig. 2:**
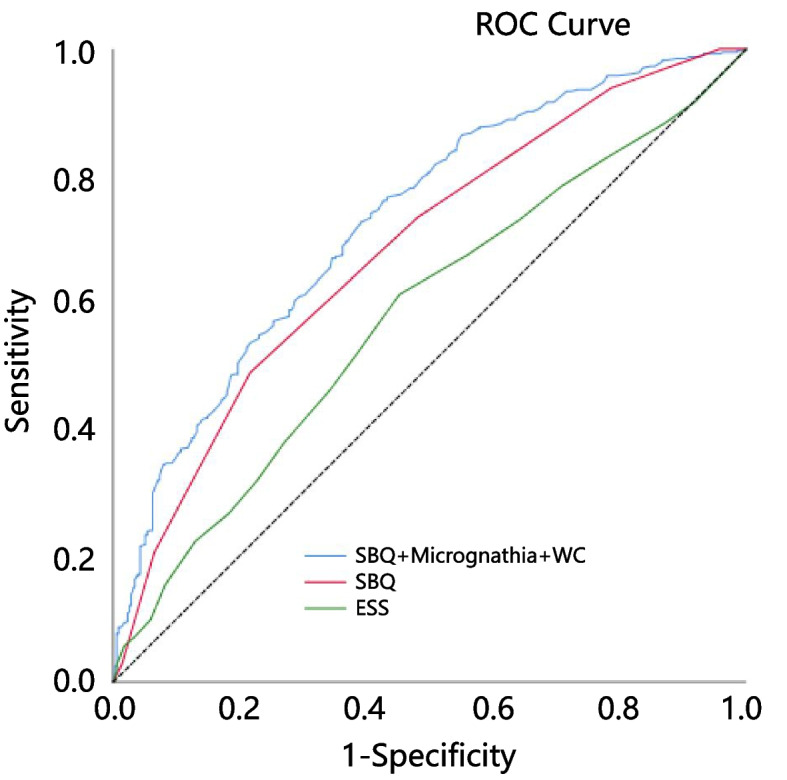
Receiver operating characteristic curve for the individual components and composite screening test. ESS: Epworth sleepiness scale; ROC: Receiver operating characteristic; SBQ: Stop-bang questionnaire; WC: waist circumference

Receiver operating characteristic curve (ROC) analysis results showed that the multivariable joint diagnosis was performed by micrognathia and WC combined with SBQ had the highest area under curve (AUC) compared to the sleep questionnaire (ESS and SBQ) as individual diagnostic index and the results showed that AUC was 0.728. Under the optimal truncation value, the sensitivity was 73%, the specificity was 61%, which was higher than the single index. When ESS was used as the diagnostic index, the AUC was 0.579, and the optimal cut-off point was 6. Under this cut-off value, the sensitivity and specificity were 67 and 44%, respectively. When SBQ was used as a diagnostic index, AUC was 0.684 and the optimal cut-off value was 4. Under this cut-off value, the sensitivity and specificity were 73 and 51% respectively. The AUC of multivariable joint diagnosis was significantly better than the AUC of ESS and SBQ (*p* < 0.0001 and *p* = 0.0002, respectively). Positive predictable values (PPV), negative predictable values (NPV), positive likelihood ratio (PLR) and negative likelihood ratio (NLR) are presented in Table 4. Therefore, combined diagnosis has more diagnostic value than single index (Fig. 2).

**Table 4:** Diagnostic accuracy for nurse-led anthropometric and oropharyngeal characteristics combined with sleep questionnaire to detect moderate/severe OSA (AHI ≥ 15)

Variables	AUC	Cut-off	Sen-	Spe-	PPV	NPV	PLR	NLR
ESS	0.579	6.00	0.67	0.44	0.57	0.54	1.20	0.72
SBQ	0.684	4.00	0.73	0.51	0.63	0.63	1.52	0.51
SBQ + Micrognathia + WC	0.729	0.49	0.73	0.61	0.68	0.66	1.88	0.44

*ESS* Epworth sleepiness scale, *NLR* Negative Likelihood Ratio, *NPV* negative predictive value, *PLR* Positive likelihood ratio, *PPV* positive predictive value, *SBQ* Stop-bang questionnaire, *Sen* Sensitivity, *Spe* Specificity, *WC* Waist circumference

### Nomogram to estimate the risk of moderate/severe OSA in patients with ACS

**Fig. 3:**
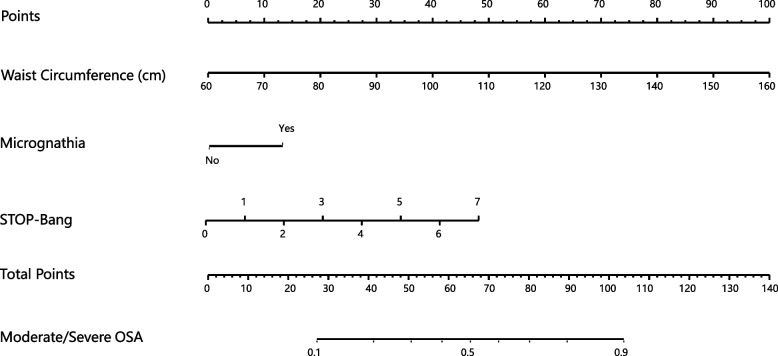
Nomogram to estimate the risk of moderate/severe OSA in Patients with ACS

A Nomogram model based on multivariate logistic regression was established with the significantly different micrognathia, waist circumference, and SBQ score as predictors in multivariate logistic analysis (Fig. 3). Using a nomogram, first find the position of each variable on the corresponding axis, then draw a vertical dotted line on the point axis, find the corresponding point, and get the total points for all variables. Finally, draw a vertical line from the total points axis to determine the moderate/severe OSA probabilities in patients with ACS.

## Discussion

In this study, a screening instrument was developed for primary screening of OSA in ACS patients. Our study found that waist circumference, micrognathia combined with SBQ improved the screening power of OSA in ACS patients, and its utility as a screening tool was confirmed by strong specificity, PPV, NPV, PLR and NLR. Furthermore, we established a simple and easy-to-use nomogram model which is integrated the 3 predictors to predict the risk of moderate/severe OSA in ACS patients. The establishment of this screening tool can refer high-risk patients with moderate/severe OSA to sleep center to relieve pressure on existing healthcare resources.

Despite the high prevalence of OSA in patients with ACS, OSA is often underdiagnosed and undertreated in cardiovascular practice [17]. Therefore, it is particularly important to develop strategies for better screening for sleep apnea in newly admitted ACS patients. Current screening strategies, such as the ESS and the SBQ, rely primarily on self-reported patient symptoms and provide mixed results. The sleep questionnaire alone has low diagnostic accuracy and is not recommended as the only diagnostic test [14, 18, 19]. The ESS is used frequently to measure excessive daytime sleepiness in research and clinical settings. We found that ESS scores were not related to moderate/severe OSA in the multivariate logistic regression model, so ESS could not predict OSA prevalence in ACS patients. Kendzerska et al. showed that ESS can be recommended for group-level comparisons, but not for individual-level comparisons due to its internal consistency [20]. ESS should not be used to evaluate the effectiveness of therapeutic interventions or to prioritize access to services [21]. The SBQ is a more reliable and easy-to-use screening tool that can stratify patients at risk for OSA based on their score. Moreover, the study by Hwang et al. showed that the SBQ could be an effective tool for screening OSA (AHI ≥ 5) in patients with cardiovascular risk factors [22]. Although the SBQ has been validated in different populations, there may still be potential biases in some validation studies due to the possible self-selection bias of patients themselves [13]. In veterans, the SBQ alone is not sufficient to confirm the presence of severe sleep apnea [23]. The SBQ also cannot rule out the presence of OSA in patients with chronic kidney disease and end-stage renal disease [24]. Consequently, to ensure reliably screening, it is recommended to validate the SBQ in a specific target population and incorporate objective indicators [25].

Previous research has shown that anthropometric characteristics, such as neck circumference, can be used as predictors of OSA severity [9]. Kim et al. used cephalometric and other variables to develop a formula to predict Koreans with suspected OSA [26]. In addition, regional obesity has also been shown to correlate with the severity of OSA, with neck fat having a direct effect on upper airway patency in women and abdominal obesity being the predominant factor in men [27]. Among middle-aged men, waist-to-hip ratio strongly predicted sleep disordered breathing in obese and nonobese men [8]. OSA is a multifactorial disease, airway collapse and poor pharyngeal muscle reactivity are one of the main pathophysiological factors [28]. Oropharyngeal crowding is a local anatomic factor, the more crowded the upper airway, the more severe the OSA. Oropharyngeal exercises can increase muscle tone, endurance, and coordination of movements in the pharyngeal and peripharyngeal muscles. Studies have shown that oropharyngeal exercises can significantly improve OSA [29]. Therefore, patient oropharyngeal parameters, such as micrognathia, upper airway, and antiadoncus, which are easily measured by nurses, should also be considered for analysis of whether they are associated with OSA severity [30].

Previous studies have shown that the visceral adiposity index (VAI) is a better predictor of clinical and coronary angiographic severity assessment in patients with ACS than other obesity indices [31]. VAI is determined by WC, BMI, fasting triglycerides (TG), and high-density lipoprotein cholesterol [32]. Compared with peripheral adiposity (BMI), central adiposity (WC) was more predictive in ACS patients [33–35]. Consistent with our research, among numerous nurse-led anthropometric parameters, only WC (OR 1.075, 95% CI 1.044–1.108, *P* < 0.001) was independently associated with the prevalence of moderate/severe OSA. Compared with no/mild OSA patients, moderate/severe OSA patients had significantly larger waist circumferences (*P* < 0.001). In addition, studies have shown that abdominal obesity, but not general obesity, seems to play a more important role in OSA [36]. Waist circumference is associated with the severity of OSA. Tom et al. suggested that waist measurements were more correlated with specific disease severity (SaO_2_ minimum and AHI) than BMI in OSA subjects [37]. These findings provide an easily measurable adjunct to assessing OSA risk. Craniofacial anomalies are common in patients with moderate/severe OSA. Both midface and mandibular hypoplasia contribute to OSA in these populations [38]. For this reason, it is important to identify these features as soon as possible. In our study, micrognathia was significantly associated with the prevalence of moderate/severe OSA (OR 2.019, 95% CI 1.020—4.000, *P* = 0.044). The study by cielo et al. showed that micrognathia was associated with more significant OSA than normal infants and that OSA improved in most infants with micrognathia after surgical correction [39].

Sleep questionnaires such as ESS and the SBQ may have recall biases, and patients may have unclear or no knowledge of their sleepiness and apnea [40]. These factors greatly decrease specificity and highlight the need for a combined-modality screening tool. The combined-modality screening tool in our study differs from these other screening tests in a number of ways. Firstly, it is specifically designed to screen moderate/severe OSA in patients with ACS. Population heterogeneity is a very important consideration, and studies have shown that arterial hypertension and neck circumference are important variables in patients with severe OSA living at high altitudes [10]. Sole et al. found that traditional OSA predictors (eg, gender, Epworth score) performed poorly in more advanced COPD patients [41]. Secondly, this tool combined physical examination characteristics and self-reported symptoms to increase sensitivity, especially in the case of atypical OSA manifestations. Friedman et al. also used a screening test combining symptoms and objective results to diagnose OSA [42]. In contrast, our tool uses a single nomogram to screen moderate/severe OSA in patients with ACS. A nomogram is simple to calculate and can be used to quickly rule out low-risk OSA [43].

If mild/no OSA patients are referred to the sleep centers for PSG can further strain existing resources, resulting in a waste of money and time. Considering the heterogeneity of patients and the need for a broader screening strategy for OSA, it is imperative to develop simpler and more reliable screening modalities to prioritize PSG treatment [44]. Our study now develops a new screening strategy that combines anthropometric and oropharyngeal measurements with SBQ, which take into account anatomical criterion. Compared with the traditional use of sleep questionnaires (ESS or SBQ) alone for screening admitted patients, the diagnostic accuracy of the new screening model (WC, micrognathia combined with SBQ) was significantly improved. After the patient is admitted to the hospital, well-trained nurses can obtain the patient's WC, presence of micrognathia and SBQ score easily, and then according to the nomogram, the probability that the patient may have moderate/severe OSA can be obtained. It may be inconvenient for the physician to obtain the patient's anthropometric and oropharyngeal parameters, but it is very convenient, accurate, and efficient to obtain the above anthropometric indicators by professional nurses. The new screening mode can not only increase the screening accuracy of OSA in ACS population, but may also improve efficiency and save medical resources. After screening, moderate/severe OSA patients with ACS can be directly referred to the sleep center for PSG.

This study had several limitations. First, this was a single-center study; Second, the nurses in the cardiology ward are unable to readily identify micrognathia based on cephalometric X-ray results (sella nasion B point angle is too small), so they may only be able to identify patients with very obvious micrognathia. In addition, judging upper airway obstruction based on images produced by doctors may affect the reliability of the results to some extent; Third, there may be intra- and inter-observer variability because anthropometric and oropharyngeal parameters were not measured multiple times in a row on the same patient, and the same patient was not measured by different nurses. Fourth, we have not conducted validation in other cohorts at present, but this will be our next research plan. Overall, the new tool performed well in evaluating OSA risk and severity in ACS patients, but this may only be a first step toward optimizing the use of sleep center resources. Fifth, cardiorespiratory polygraphy of patients using portable sleep monitors may underestimate the severity of OSA. However, studies have shown that portable polygraphy can be an alternative to polysomnography in diagnosing OSA [45].

## Conclusions

In brief, we present a new tool for OSA screening among ACS patients by combining WC, micrognathia, and SBQ, which has greater power than single questionnaires or measurements. We propose that the nurse-led multi-modality screening tool be used to evaluate OSA risk and severity in ACS patients for timely diagnosis and better treatment.

## Data Availability

Data generated or analyzed during this study are included in this published article.

## Abbreviations

ESS: Epworth sleepiness scale
NLR: Negative Likelihood Ratio
NPV: Negative predictive value
PLR: Positive likelihood ratio
PPV: Positive predictive value
SBQ: STOP-BANG Questionnaire
Sen-: Sensitivity
Spe-: Specificity
WC: Waist circumference
